# Mission analysis, dynamics and robust control of an indoor blimp in a CERN detector magnetic environment

**DOI:** 10.3389/frobt.2023.1238081

**Published:** 2023-10-13

**Authors:** Francesco Mazzei, Lorenzo Teofili, Fabio Curti, Corrado Gargiulo

**Affiliations:** ^1^ Automation Robotics and Control for Aerospace (ARCA) Laboratory, School of Aerospace Engineering, University of Rome La Sapienza, Rome, Italy; ^2^ Detector Mechanics, Experimental Physics (EP) Department, European Organization for Nuclear Research (CERN), Geneva, Switzerland; ^3^ Department of Systems and Industrial Engineering, The University of Arizona, Tucson, AZ, United States

**Keywords:** robotic systems, CERN particle detectors, harsh environment, aerial inspection and mapping, unmanned aerial vehicle, indoor blimp, magnetic disturbances, robust control techniques

## Abstract

At the European Organization for Nuclear Research (CERN), a Research and Development (R&D) program studies robotic systems for inspection and maintenance of the next-generation of particle detectors. The design and operation of these systems are affected by the detector’s cavern harsh environment consisting of high magnetic fields and radiations. This work presents a feasibility study for aerial inspection and mapping around a CERN particle detector using a robotic Lighter-than-Air (LtA) Unmanned Aerial Vehicle (UAV), specifically a blimp. Firstly, mission scenarios and the detector environment are introduced; in this context a new empirical model is proposed for the estimation of magnetic disturbances resulting from the interaction of electromagnetic motors with the external magnetic field. Subsequently, the design of a reference blimp and the control system is presented, comparing different control techniques, namely, Computed Torque Control (CTC), Sliding Mode Control (SMC) and Nonsingular Terminal Sliding Mode Control (NTSMC). Finally, the results of trajectory tracking simulations are reported, considering both the uncertainties of the dynamic parameters and the estimated magnetic disturbances. This work demonstrates that the blimp successfully follows desired trajectory, navigating complex environments while maintaining stability and accuracy. Despite the challenges posed by high magnetic fields, indoor blimps can effectively offer safer and more efficient approaches to facility surveillance and maintenance, reducing radiation exposure for human personnel and minimizing detector downtime.

## 1 Introduction

Over the past few years, there has been a significant rise in the deployment of robots to operate in challenging and hazardous environments (Wong et al., 2017). They have proven to be helpful in fields such as nuclear decommissioning Seward and Bakari (2005), offshore maintenance (Khalid et al., 2022), space exploration (Gao and Chien, 2017), and deep mining (Marshall et al., 2016). The utilization of robots in these environments can prevent people from working in dangerous places and increase productivity in high-risk and unstructured facilities.

In this context, the European Organization for Nuclear Research^1^ (CERN) faces the challenge of designing robots to use in semi-structured and harsh environments such as the underground caverns that host the CERN particle detectors and all their related working equipment.

The main particle collider at CERN, the Large Hadron Collider^2^ (LHC), currently has four particle detectors (which are an ensemble of sensors needed to study the results of particle beam collisions): ATLAS^3^, ALICE^4^, CMS^5^, and LHCb^6^. These detectors are hosted in underground caverns, which are industrial environments^7^ characterized by high magnetic fields and radiation.

The complex and delicate equipment in these sites requires frequent inspections, maintenance, and upgrades, which are currently carried out by human personnel. However, the next-generation of particle detectors will present a radiation environment that will be a threat to human presence even when the beam is not running (Abada et al., 2018). In some critical areas, personnel would not be permitted to work for more than a few hours per year to limit the absorbed radiation doses. This would impact work continuity and make time-consuming activities in those areas practically unfeasible.

To guarantee the safety of its personnel and at the same time ensure inspections, maintenance, and upgrade work on detectors, the CERN Experimental Physics (EP) Detector Technologies (DT) department^8^ has started an R&D activity^9^ to study (among other topics) the integration and use of robotic and automatic systems in the detector caverns for future experiments.

Robotics and automation could be particularly useful in the underground caverns of detectors, providing access even when the detector is on (for security and radiation reasons no personnel is allowed inside). This would enable immediate verification of triggered alarms without the need to turn off the detector. Furthermore, these system can also perform repetitive and time-consuming tasks such as continuous data acquisition.

In this context, this paper presents two mission scenarios for an indoor blimp in a reference future particle detector environment. The first scenario is a visual inspection of the detector (typical in case of alarm triggering), while the second focuses on the acquisition of 3D maps of environmental data (such as radiation levels, magnetic fields, temperature, or humidity) in the cavern. The reference detector environment is discussed in Section 2.1, and the mission scenarios are discussed in detail in Section 2.2. Section 2.3 shows the magnetic environment that characterises the reference detector cavern in which the UAV system will have to fly. The choice of the blimp over other UAVs (such as drones) is due to its intrinsic safety. In case of failure, the buoyancy force guarantees slow vertical motion (ascending or descending), avoiding the system from crushing over delicate equipment; this choice is further addressed in Section 2.4. The design is powered by electromagnetic motors because they are the only actuators that can ensure a light, compact, and agile design while considering their power source. However, these motors are susceptible to external magnetic fields, and therefore, this paper investigates the feasibility of controlled flight in an operational scenario characterized by a non-negligible background magnetic field. In particular, this work proposes a semi-empirical model to predict the disturbance torques and forces acting on the electromagnetic motors due to the external magnetic field (Section 2.5).

The detailed design of the blimp is presented in Section 3.1 while in Section 3.2 the mathematical model of the equations of motion of the system is given. In Section 3.3 the robust control system techniques are reported for the trajectory tracking control simulation including uncertainties in dynamic parameters and magnetic disturbances.

In Section 4, the simulated results of non-linear robust trajectory tracking for two mission scenarios are presented and compared. The simulated missions are repeated for different control techniques: Computed Torque Control (CTC), Sliding Mode Control (SMC) and Nonsingular Terminal Sliding Mode Control (NTSMC). Finally, the conclusions and future work are reported in Section 5.

## 2 Mission analysis

### 2.1 The reference detector environment: the FCC detectors

This work is based on the Future Circular Collider (FCC)^10^ ^,^ ^11^ which is the CERN next-generation particle collider project in collaboration with the international scientific community. At the writing time, there are different concurring designs for the FCC collider that result in different magnetic and radiation environments within the detector cavern. There are three main FCC designs that have been proposed:

1. FCC-hh: hadron collisions (proton–proton and heavy ion) Abada et al. (2018).
2. FCC-ee: electron–positron collisions Abada et al. (2019a);
3. FCC-he: proton-electron collisions or proton-heavy ion collisions Abada et al. (2019b).

The results presented in this paper have been obtained by considering the FCC-hh detector cavern environment Abada et al. (2018). This is not a loss of generality because, by design, it presents the most challenging radiation environment and imposes the strictest requirements for robotics in terms of magnetic fields. Therefore, a device that meets the mission requirements for FCC-hh should also be suitable for similar missions in FCC-ee and FCC-he.

**FIGURE 1:**
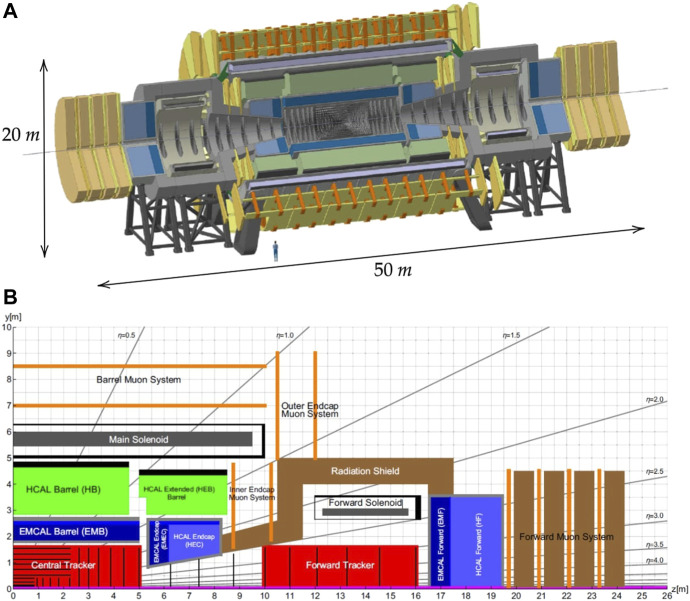
**(A)** 3D representation of the FCC particle detector. The human figure in the image is included to provide a sense of scale. The detector has a diameter of approximately 20 m and a length of 50 m. **(B)** longitudinal section with dimensions of the FCC-hh reference detector. The components are indicated. The section represents one-quarter of the entire detector, the other detector parts and dimensions can be obtained by symmetry. From Abada et al. (2018).

The layout of the FCC-hh reference detector is taken from Abada et al. (2018) and shown in Figure 1. In the top image a 3D scale representation gives the real size of the detector which has dimensions similar to ATLAS, with a diameter of around 20 m and a length of around 50 m. In the bottom image a longitudinal cross section of the detector is shown with the principal detector components highlighted. At the z = 0, y = 0 position the collision point is located. In grey are visible the 4 T main and forward solenoids. They create the magnetic field to bend the trajectories of charged particles for accurate particle identification. The trackers (in red), map the trajectory of the particles resulting from the collisions. The calorimeters (including electromagnetic calorimeters for electrons and photons, in bluem, and hadronic calorimeters for hadrons, in green) indicated with the suffix CAL, absorb the collisions resulting particles and measure their energy. The muon systems (in orange) detect and study muon particles resulting from the collisions, that cannot be captured by calorimeters. The symbol *η* represents the pseudorapidity parameter, a tool in colliding beam experiments for quantifying angles relative to the axis of the beam collision. It assumes a value of 0 when describing particle paths perpendicular to the beam axis, while adopting positive or negative values to denote trajectories inclined at an angle to the beam. Finally, the radiation shield (in brown) protect sensitive detector components and personnel from harmful radiation produced during high-energy particle collisions.

**FIGURE 2:**
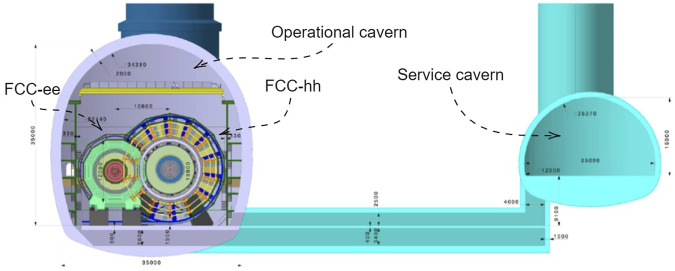
Cross section of the 3D model of the FCC-hh detector and FCC-ee detector situated within the operational detector cavern and the service cavern. The dimensions reported in the figures are in millimeters. The Fcc-ee and FCC-hh are shown in the operational cavern at the same time only for illustration purposes, in reality, if built, they will be installed in the cavern in different periods. Image taken From Abada et al. (2018).

The FCC-hh detector is situated in an operational cavern of approximately 35 m × 35 m × 100 m and connected to a service cavern where racks, water pumps, and electric power generators are located. Figure 2 shows the FCC-hh and FCC-ee detector inside the operational cavern and the service cavern.

### 2.2 Mission scenarios and trajectory generation strategy

Currently, operators regularly perform visual inspections of the detectors at fixed intervals to ensure their proper functioning. They also conduct on-demand surveys if any anomalies, such as high temperature or water/gas leaks, are identified. Although this task is crucial, it is not complex and could be carried out by robots to prevent humans from being exposed to high radiation doses.

Furthermore, in a particle detector is also important to monitor the environment in the operational and service cavern. This can be done by measuring the magnetic fields, radiations, temperature, humidity, pressure, or gas concentration in the cavern. At the moment these quantities are measured with sensors positioned at fixed points. A moving robotic system would allow the acquisition of these measurements in a higher number of points that could also be arbitrarily located in areas where a sensor cannot be installed permanently. This could provide a very refined measurement grid opening the door to several applications. For instance, having a fine grid of the actual radiation environment in the cavern would allow for optimizing the path for a human intervention to guarantee the minimum absorbed dose. Furthermore, a fine grid of temperature data may reveal unexpected hot zones that would need further inspection and the acquisition of gas concentration data could reveal leaks in the service pipes in specific cavern sectors. Thus, data acquisition is another crucial task to guarantee the correct functioning of the detector, however, it is a repetitive and time-consuming one, which makes it suitable to be undertaken by robots automatically.

It is clear that the best robotic system to perform this kind of tasks is an Unmanned Aerial Vehicle (UAV) since it can easily move in 3 dimensions avoiding obstacles and reaching points impossible to reach for non-flying robots. In this context, this paper presents two mission scenarios for a UAV in the reference future particle detector environment:

1. The visual inspection of the detector.
2. The acquisition of 3D maps of environmental data in the detector cavern.

Two trajectories have been designed for the UAV to fulfill the requirements of the two mission scenarios, which are presented in Section 4. The inspection trajectory has been designed to be the shortest path that allows for a complete inspection of the detector from one side to the other. The 3D mapping trajectory has been generated to ensure that the UAV accurately spans a specific volume and acquires data at the maximum number of points before the battery needs recharging.

Both trajectories consider the actual dimensions of the detector and are based on the quintic polynomials method for a path with via points Craig (2005). The method has been implemented in MATLAB^®^ ^12^ using a built-in function. Both trajectories make the UAV fly through areas with the strongest magnetic fields to test the effectiveness of the control system against disturbances that occur when crossing different magnetic field regions. 

### 2.3 The reference magnetic environment

The interaction point is at the core of a particle detector, where accelerated particle beams collide and generate resultant particles that physicists analyze using the detector’s sensors. A uniform, time-invariant magnetic field around the interaction point is crucial for analyzing the collision’s results. In the reference detector, this magnetic field is generated by three solenoids capable of producing a 4 T field in their inner part. The main solenoid has a diameter of approximately 10 m and a length of 20 m, while the other two are forward solenoids with a diameter of 5 m. A schematic of the reference detector’s internal components that highlights the solenoids’ positions and dimensions in the detector is shown in the bottom image of Figure 1. The FCC-hh detector lacks electromagnetic shielding, and its electromagnets produce a time-invariant, non-uniform stray magnetic field in the detector’s operational cavern.

**FIGURE 3:**
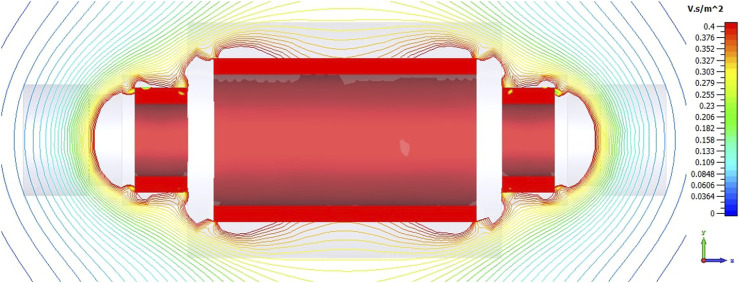
Magnetic field simulation of the FCC magnet system. The stray fields ranging from about 300 mT just outside the detector to 150 mT at the cavern walls.

To acquire simulation data for the magnetic field within the operational FCC-hh detector cavern, a simplified version of the detector was modeled using CST Studio Suite^13^, as depicted in Figure 3. The representation of the detector was streamlined to encompass only the structure of the three solenoids responsible for generating the magnetic field, omitting all other components illustrated in Figure 1. The simulated map reveals that the UAV flying in the cavern will be exposed to stray fields ranging from approximately 300 mT just outside the detector to 150 mT at the cavern walls.

### 2.4 Choice of the specific UAV: a blimp

In Section 2.2, the mission scenarios have been presented and a UAV has been indicated as the best choice to satisfy the mission requirements. In this section, a quick analysis of the currently available UAVs is made and the reason behind the choice of a blimp is given. UAV systems can be classified into four categories Nonami et al. (2010):

1. Fixed-wing UAVs: airplanes.
2. Rotary-wing UAVs: helicopters or multi-rotors.
3. Lighter-than-Air (LtA) UAVs: blimps and balloons.
4. Flapping-wing UAVs: robots flying insects.

By analyzing the various types of flying robots, fixed-wing UAVs are more suitable for covering long distances in outdoor applications, as they have a higher range and endurance compared to other types of UAVs. Rotary-wing UAVs, such as quadcopters, are used when greater maneuverability and stability are required, especially in indoor spaces where they can hover and fly in confined areas. Finally, LtA UAVs are used both for outdoor and indoor applications when speed and maneuverability are not required, as they are slower but can stay in the air for longer periods of time. Currently, flapping UAVs are still in an experimental phase with few practical applications, and are mainly used for research purposes.

The choice of using LtA UAVs, specifically indoor blimps, at CERN is based on several advantages that they offer over other types of UAVs. The primary benefit is related to safety concerns, as blimps are much safer in the event of a failure compared to other UAVs that may fall and damage expensive equipment. Blimps can slowly descend or rise upwards due to the relationship between gravity and buoyancy force, reducing the risk of causing damage. Another advantage of LtA UAVs is their ability to provide a longer operational time with a single battery recharge than other UAVs with the same battery capacity and payload. This is because blimps can float in the air with minimal energy consumption, which can be particularly useful for long inspection or monitoring missions.

In recent years, LtA UAVs have been gaining attention in both military and research aerial robotics due to their promising potential for many applications Sebbane (2012); Zheng et al. (2021). Indoor blimps have been developed for infrastructure inspection Nitta et al. (2017), environmental data collection Kantor et al. (2001), indoor localization and mapping Müller and Burgard (2013), education and research platforms Gorjup and Liarokapis (2020), vision-based human-robot interaction Yao et al. (2017), and other activities. Researchers continue to explore the possibilities and capabilities of LtA UAVs, making them an exciting field of study in aerial robotics. 

### 2.5 Magnetic disturbance

After choosing the type of UAV to use, it is important to consider that these systems are almost always equipped with Electromagnetic Motors (EMs) as the propulsion system. These types of motors can interact with the surrounding magnetic field present inside the cavern. This interaction then generates disturbances on the system, such as force and torque generation.

The external stray magnetic field of the detector can affect the EMs in two ways. Firstly, if the external magnetic field is strong enough, it can interfere with the internal functional magnetic fields of the motors and even cause them to stop working properly. Secondly, the external magnetic field can exert forces and torques on the electromagnetic actuators, and therefore on the entire blimp, leading to disturbances in the motion that could potentially prevent the system from following the desired trajectory.

This paper makes the strong assumption that the motors will continue to function as expected even in the presence of an external magnetic field, thus ignoring the first type of disturbance. However, it considers the forces and torques acting on the motors due to the magnetic field. In this section, a novel mathematical model used to quantify this disturbance, denoted as ***τ***_*d*_, will be presented and discussed.

Typically, brushed EMs used in blimps are constructed with a holed permanent magnet enclosed in a ferromagnetic cylindrical cover, with the motor axis as its axis. The majority of the magnetic field generated by the permanent magnet closes the path flowing inside the cover due to the high magnetic permeability of its material, leaving a low magnetic field outside the motor. Inside the permanent magnet hole, the copper winding is free to rotate.

These components are made of different materials: the permanent magnet is usually made of hard ferromagnetic material, while the cylindrical cover is made of soft ferromagnetic material. Soft materials change their magnetization even when exposed to weak external magnetic fields ***B***, while hard materials require much higher external magnetic fields to modify their magnetization. Copper, on the other hand, is not ferromagnetic and does not interact with the external magnetic field.

**FIGURE 4:**
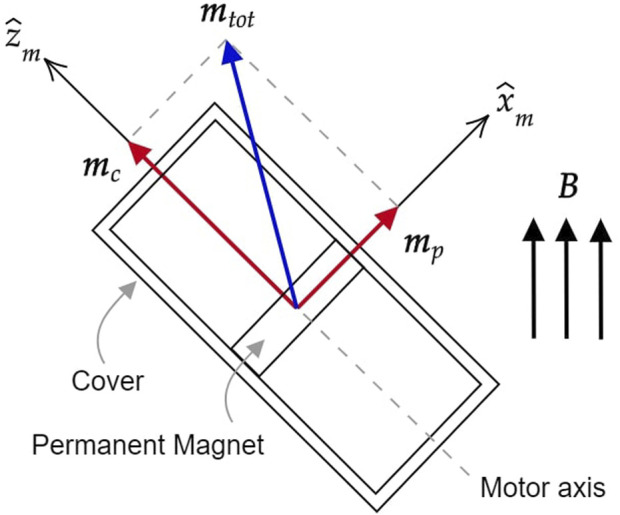
Geometrical modeling of the motor within a uniform magnetic field ***B*** with the magnetic dipole moments associated with the rotor, the stator and the total sum of the two.

In this paper, the motor is modeled as a magnetic dipole moment ***m***_*tot*_, which interacts with the ***B*** field and generates the disturbance ***τ***_*d*_ in terms of forces and torques. As shown in Figure 4, the total dipole moment ***m***_*tot*_ is the sum of two magnetic dipole moments. The former, indicated as ***m***_*p*_, is the one of the Permanent Magnet (PM) which, being a permanent magnetized ferromagnetic materials, can thus be represented as magnetic dipole. The latter, indicated as ***m***_*c*_, is the magnetic dipole moment generated by the magnetization of the cover soft ferromagnetic material in the presence of the local external field. The PM dipole ***m***_*p*_ is always perpendicular to the axis of rotation of the motor and its absolute value can be obtained from the motor manufacturer. Therefore, in the proposed model the ***m***_*p*_ is considered to be constant and independent of the external magnetic field ***B***. This assumption is justified by the fact that the motor PM, already magnetized, would need very high magnetic field to change its absolute value and orientation. The cover dipole moment ***m***_*c*_ instead, originated from the magnetization of the soft material induced by the presence of the external field ***B***, strongly depends on the orientation of the motor axis with respect to the field and on the size of the field itself. In this work, the absolute value of ***m***_*c*_ is considered to be constant while the orientation changes in such a way that the cover dipole is always aligned with the ***B*** field lines. To obtain the absolute value of the two dipoles shown in Figure 4, this motor model has been simulated with CST Studio Suite as immersed in a constant 0.3 T external magnetic field (the maximum value in which the blimp has to fly). Varying the angles of the motor axis with respect to the magnetic field and considering the worst-case scenario, the highest absolute value of ***m***_*c*_ resulted is taken as a reference. Referring to Figure 4 and indicating the motor axis as ${\hat{z}}_{m}$ , the process of alignment of the ***m***_*c*_ dipole with the external field lines, during the blimp motion in space, is modelled as follows:

$$m_{\mathrm{tot}}=\left\{\begin{array}{cc} m_{p}+m_{c}\; & \text{if}\;B\cdot \hat{z}>0\\ m_{p}\; & \text{if}\;B\cdot \hat{z}=0\\ m_{p}-m_{c}\; & \text{if}\;B\cdot \hat{z}<0 \end{array}\right.$$ (1)

With this formulation, the motor total dipole moment turns out to be the sum of the PM dipole (always constant in module and orientation) and the cover dipole (constant in module but not in direction) with the latter aligning itself with the external field during the movement of the motor system.

Once the total motor magnetic dipole is known, the disturbance ***τ***_*d*_, comes from the force ***f*** and the torque ***c*** of a generic magnetic dipole ***m*** in a magnetic field ***B*** (*x*, *y*, *z*) Griffiths (2017); Stöhr and Siegmann (2006):

$$\tau_{d}^{m}=\left[\begin{array}{c} f_{d}^{m}\\ c_{d}^{m} \end{array}\right]=\left[\begin{array}{c} \nabla \left(m_{\mathrm{tot}}\cdot B\right)\\ m_{\mathrm{tot}}\times B \end{array}\right]$$ (2)

It is worth concluding this section by discussing the assumption made at its beginning, that is the electric motor will continue to work as expected also in an external magnetic field, and the implication of not adopting it. To non-negligibly change the motor functioning, an external magnetic field should be strong enough to saturate the cover, penetrate inside it and modify the magnetization of the hard ferromagnetic permanent magnet of the motor by hysteresis. For this feasibility study, this has been assumed as a non-primary effects, since it can always be limited by further shielding the electromagnetic motor (with *μ* metals for instance), and priority has been given to the study of the forces acting on the motors. However, this assumption should be better investigated in future developments. Indeed, if the external magnetic field is strong enough to sensibly modify the residual magnetization of the motor’s permanent magnet by hysteresis, it may impact the performance of the controllers proposed in this study. In such a case, the motor will not follow anymore its nominal current-torque curve. If the residual magnetization of the permanent magnet is decreased by the external magnetic field, the motor will require more current to provide the same torque that nominally is delivered for a lower current value, decreasing the battery life, and so the mission duration. Furthermore, if the residual magnetization is excessively decreased the motor may not be able to provide the needed torques in operations, also if it can deliver them nominally. Another impact of the hysteresis phenomenon will be on the forces acting on the motor. This will result in location-dependent variations in the magnitude and orientation of the dipole ***m***_*c*_ in the presented model, causing further disturbances in the system. Since hysteresis results in nonlinear and often asymmetric changes in magnetization, precise quantification of the perturbations will require further analysis, with the use of experimental data and more detailed simulations.

## 3 System modeling

### 3.1 Blimp reference design

The detector cavern environment introduced in Section 2 presents several challenges, such as narrow passages and the presence of a residual magnetic field. To address these issues, the design of the blimp system was based on a set of both design and functional requirements, aiming to create a system capable of operating and moving effectively in the harsh environment of the detector cavern. Table 1 outlines the essential design requirements and specifications for the proposed blimp system. It includes dimensions, mass budget, payload details, sensor capabilities, controller accuracy, communication setup, navigation methods, magnetic environment considerations, reliability measures, and operational duration.

**TABLE 1:** Blimp design requirements and specifications.

Requirement	Value/Element	Rationale
Dimensions	1.5 × 0.6 × 0.6 m	The width and height dimensions are selected to allow the blimp to pass through the minimum size of a narrow passage such as a door, the length to allow agile manoeuvring
Mass budget	450 g	After sizing, the buoyancy force that the helium balloon is capable of providing is determined, and thus the weight that counterbalances the buoyancy. This value considers the mass of the balloon, helium, motors and payload
Payload mass	200 g	This value includes on-board electronics and sensors
Payload sensors	Magnetic field, radiation, temperature, pressure, humidity	To allow data collection capabilities. All masses shall fall within the 200 g limit. For radiation and magnetic field sensors, internally developed built-in solutions from CERN will be considered Stifter et al. (2016); Teyssier et al. (2011)
Controller accuracy	1 cm (position) and 1° (orientation)	To ensure that the system can navigate complex environments safely and efficiently, avoiding collisions and enabling precise inspection and mapping tasks
Communication	WiFi board	The WiFi board, connected to the CERN network, serves for two purposes: to upstream the navigation data to the blimp and to downstream the blimp log, telemetry and payload data to the ground station
Navigation	Indoor positioning system (motion capture)	The use of onboard LiDAR may encounter challenges due to laser refraction on cavern materials. However, the blimp shall be equipped with an onboard navigation system (IMU) to navigate when a WiFi connection is absent
Magnetic environment	0.3 T	Based on CST simulations on the FCC-hh detector, the EM motors shall ensure stable and controlled flight against magnetic forces and torques on them
Duration	30 min	To allow for the successful completion of inspection and mapping missions, enabling optimal data acquisition, all without the need for recharging the batteries

**FIGURE 5:**
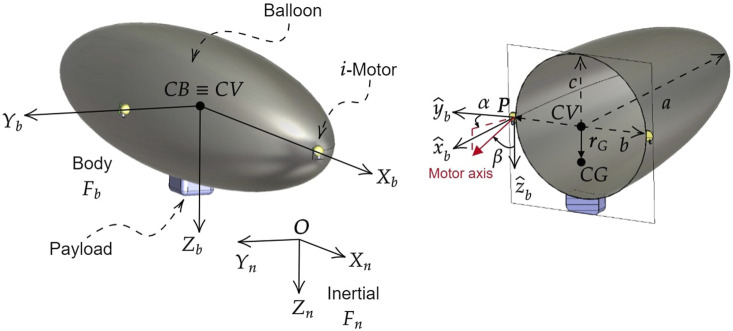
Reference frames for the blimp. The inertial frame *F*_*n*_ has its origin ***O*** at the arbitrary point and its axes *X*_*n*_, *Y*_*n*_, *Z*_*n*_ aligned with the North-East-Down directions. The body frame *F*_*b*_ has its origin at CV of the blimp, coincident with the CB, and its axes *X*_*b*_, *Y*_*b*_, *Z*_*b*_ always directed as the principal inertia axes of the ellipsoidal balloon with *Z*_*b*_ pointing towards the gondola. The position of the motors is given by ***P*** and their orientation angles *α*_*i*_ and *β*_*i*_. The blimp design shows its ellipsoidal balloon with semi-axes a, b, and c, and the gondola located at the bottom of the balloon which houses the payload, energy source, and control electronics.

Based on these requirements, the blimp used as a reference for this study is composed of an ellipsoidal balloon filled with helium to generate buoyancy, with semi-axes *a*, *b*, and *c*, as shown in Figure 5. A gondola is located on the bottom part of the balloon, which hosts the payload, energy source (a battery), and control electronics. Based on the work of Aman (2021), the blimp reference model is controlled by eight motors positioned on the balloon surface, allowing for full control of the platform. The reference values of the blimp design parameters considered for the simulation are shown in Table 2. To model the motors, commercially available motors for UAVs have been used, specifically brushed coreless DC-motors^14^, with a 4 cm fan. The balloon geometry was designed to meet the design requirements, with specific values for its semi-axes, length of 1.70 m and diameter of 0.64 m, as shown in Figure 5 and Table 2.

**TABLE 2:** Blimp design parameters.

Parameter	Value	Description
*a*	0.85 m	Ellipsoid *x* semi-major axis
*b*	0.32 m	Ellipsoid *y* semi-minor axis
*c*	0.32 m	Ellipsoid *z* semi-minor axis
*V*	0.70 m^3^	Balloon volume
*m*_*hull*_	0.09 kg	Balloon mass
*m*_*motor*_	0.03 kg	Single motor mass
*m*_*gondola*_	0.20 kg	Gondola mass
*m*_*total*_	0.45 kg	Total blimp mass
*f*_*B*_	4.38 N	Buoyancy force at room temperature
*f*_*m*_	±0.1 N	Saturation thrust generated by the motor + fan along the motor axis
*m*_*p*_	0.5 Am^2^	Permanent magnet dipole
*m*_*c*_	0.1 Am^2^	Motor cover dipole

In Figure 5 the two reference frames in which the blimp motion is studied are reported: the inertial frame *F*_*n*_ with arbitrary origin $O={\left[\begin{array}{ccc} 0 & 0 & 0 \end{array}\right]}^{T}$ in $R^{3}$ and the *X*_*n*_, *Y*_*n*_ and *Z*_*n*_ axes aligned in the North-East-Down (NED) directions; the body frame *F*_*b*_, with origin at the blimp Center of Volume (CV), coincident with the Center of Buoyancy (CB), and its *X*_*b*_, *Y*_*b*_ and *Z*_*b*_ axes always directed as the principal inertia axes of the ellipsoidal balloon with the *Z*_*b*_ pointing towards the gondola.

With reference to Figure 5, for the *i*th motor attached to the balloon, the position vector from the center of volume of the blimp is denoted with ***P***_*i*_, and its components, expressed in the body frame *F*_*b*_ are labeled *x*_*pi*_, *y*_*pi*_, *z*_*pi*_. The orientation of the *i*th motor axis (the axis in which the motor can provide thrust) with respect to the body frame *F*_*b*_ is given by the angles *α*_*i*_ and *β*_*i*_. For the sake of clarity, a reference system with axes *x*_*b*_, *y*_*b*_ and *z*_*b*_ oriented as the reference system *F*_*b*_ and with origin in the *i*th motor position ***P***_*i*_ has been represented in Figure 5 right to visualize the angles *α*_*i*_ and *β*_*i*_. The positions and orientations of the motors of the reference blimp on its balloon in the body frame are reported in Table 3.

**TABLE 3:** Position and orientations of the eight motors of the reference blimp in the body frame *F*_*b*_. This configuration allows the blimp to deliver forces and torques with no coupling on each body axis.

*x*_*pi*_ [m]	*y*_*pi*_ [m]	*z*_*pi*_ [m]	*β*_*i*_ [deg]	*α*_*i*_ [deg]
*a*	0	0	0	0
*a*	0	0	90	90
−*a*	0	0	90	90
−*a*	0	0	0	0
0	*b*	0	0	0
0	*b*	0	90	0
0	−*b*	0	0	0
0	−*b*	0	90	0

The inertia matrix *I*_*RB*_ and the position of the center of gravity *CG* with respect to the center of volume *CV*, denoted as ***r***_*G*_ in Figure 5, were estimated in the body frame considering the motors and the gondola as point masses, while the balloon was considered as a mass uniformly distributed over a surface. This led to the following expression of the inertia matrix and center of gravity position for the reference blimp:

$$I_{RB}=\left[\begin{array}{ccc} 0.032 & 0 & 0\\ 0 & 0.076 & 0\\ 0 & 0 & 0.059 \end{array}\right]\text{kg}\cdot {\text{m}}^{2}$$ (3)

$$r_{G}=\left[\begin{array}{c} 0\\ 0\\ 0.15 \end{array}\right]\text{m}$$ (4)

These values are the ones used to obtain the results reported in Section 4.

### 3.2 Mathematical modelling

This section introduces and discusses the mathematical model used to describe the behaviour of the small indoor blimp, which is a simplified form derived from the non-linear airship model Bestaoui and Hima, (2002); Yamasaki and Goto (2003); Zufferey et al. (2006). It is assumed that the blimp behaves as a rigid body, meaning that the balloon does not undergo any deformation due to pressure or temperature variation. This assumption is justified since the blimp is intended for indoor use, where the environment is less affected by atmospheric perturbations such as temperature changes or wind.

The vector describing the instantaneous linear and angular velocities of the blimp in the body frame *F*_*b*_ is $\xi ={\left[\begin{array}{cc} {\left(v\right)}^{T} & {\left(\omega \right)}^{T} \end{array}\right]}^{T}$ with $v={\left[\begin{array}{ccc} v_{x} & v_{y} & v_{z} \end{array}\right]}^{T}$ and $\omega ={\left[\begin{array}{ccc} \omega_{x} & \omega_{y} & \omega_{z} \end{array}\right]}^{T}$ while the vector describing the position and orientation of the blimp with respect to the inertial frame *F*_*n*_ is $\eta ={\left[\begin{array}{cc} {\left(\eta_{1}\right)}^{T} & {\left(\eta_{2}\right)}^{T} \end{array}\right]}^{T}$ with $\eta_{1}={\left[\begin{array}{ccc} x & y & z \end{array}\right]}^{T}$ and $\eta_{2}={\left[\begin{array}{ccc} \phi & \theta & \psi \end{array}\right]}^{T}$ where *ϕ*, *θ* and *ψ* are roll, pitch and yaw angles, respectively. The kinematic equation relating the linear and angular velocities is given by:

$$\dot{\eta }=J\left(\eta \right)\xi$$ (5)

where, $J\left(\eta \right)\in R^{3\times 3}$ is the kinematics transformation matrix expressed as

$$J\left(\eta \right)=\left[\begin{array}{cc} J_{1}\left(\eta_{2}\right) & 0\\ 0 & J_{2}\left(\eta_{2}\right) \end{array}\right]$$ (6)

where, ***J***_1_(***η***_2_) and $J_{2}\left(\eta_{2}\right)\in R^{3\times 3}$ are the rotation matrix from the body frame *F*_*b*_ to the inertial frame *F*_*n*_ and the transformation matrix from angular velocities to attitude angle rates, respectively Fossen (1994).

In literature, the 6 degree-of-freedom (DoF) nonlinear dynamic equations in the body reference frame *F*_*b*_ are typically derived using the Newton-Euler method. To accomplish this, the design of the airship is often likened to that of an underwater vehicle, as seen in works such as Zufferey et al. (2006); Fossen (1994); Gomes (1990); Sagatun and Fossen (1991). These equations are presented below:

$$M\dot{\xi }+C\left(\xi \right)\xi +D\left(\xi \right)\xi +g\left(\eta \right)=\tau$$ (7)

where, $M\in R^{6\times 6}$ is the intertia matrix including added mass terms; ***C*** (***ξ***) is the Coriolis and centripetal matrix including added mass terms; ***D*** (***ξ***) is the damping matrix; ***g*** (***η***) is the vector of restoring forces and moments; ***τ*** is the control vector. Eq. 7 is an explicit function of ***ξ*** and, in order to asymptotically follow a desired trajectory defined as a time series of both position and orientations in the inertial frame *F*_*n*_, it must be an explicit function of ***η*** Yang et al. (2012); Bagheri and Moghaddam (2009):

$$M_{\eta }\left(\eta \right)\ddot{\eta }+C_{\eta }\left(\xi ,\eta \right)\dot{\eta }+D_{\eta }\left(\xi ,\eta \right)\dot{\eta }+g_{\eta }\left(\eta \right)=\tau_{\eta }$$ (8)

where the following kinematic transformations [assuming that ***J*** (***η***) in Eq. 5 is non-singular Londhe et al. (2017)] are applied

$$\begin{array}{cc} M_{\eta }\left(\eta \right)\; & =\;J^{-T}\left(\eta \right)MJ^{-1}\left(\eta \right)\\ C_{\eta }\left(\xi ,\eta \right)\; & =\;J^{-T}\left(\eta \right)\left[C\left(\xi \right)-MJ^{-1}\left(\eta \right)\dot{J}\left(\eta \right)\right]J^{-1}\left(\eta \right)\\ D_{\eta }\left(\xi ,\eta \right)\; & =\;J^{-T}\left(\eta \right)D\left(\xi \right)J^{-1}\left(\eta \right)\\ g_{\eta }\left(\eta \right)\; & =\;J^{-T}\left(\eta \right)g\left(\eta \right)\\ \tau_{\eta }\left(\eta \right)\; & =\;J^{-T}\left(\eta \right)\tau \end{array}$$ (9)

If external disturbances and parameter uncertainties are considered, Eq. 8 becomes:

$$\begin{array}{cc} & \left({\hat{M}}_{\eta }\left(\eta \right)\ddot{\eta }+\Delta M_{\eta }\left(\eta \right)\right)\ddot{\eta }+\left({\hat{C}}_{\eta }\left(\xi ,\eta \right)+\Delta C_{\eta }\left(\xi ,\eta \right)\right)\dot{\eta }\\ & \;+\left({\hat{D}}_{\eta }\left(\xi ,\eta \right)+\Delta D_{\eta }\left(\xi ,\eta \right)\right)\dot{\eta }+{\hat{g}}_{\eta }\left(\eta \right)+\Delta g_{\eta }\left(\eta \right)=\tau_{\eta }+\tau_{d} \end{array}$$ (10)

where ${\hat{M}}_{\eta }\left(\eta \right)$ , ${\hat{C}}_{\eta }\left(\xi ,\eta \right)$ , ${\hat{D}}_{\eta }\left(\xi ,\eta \right)$ and ${\hat{g}}_{\eta }\left(\eta \right)$ are the known values (approximated model parameter values) of the blimp; Δ ***M***_***η***_(***η***), Δ ***C***_***η***_(***ξ***, ***η***), Δ ***D***_***η***_(***ξ***, ***η***) and Δ ***g***_***η***_(***η***) are the parameter uncertainty values and ***τ***_*d*_ is the vector of external disturbances defined in Section 2.5. Parameter uncertainties and external disturbances in Eq. 10 can be collected into a single term known as *lumped uncertainty* Δ ***f***

$${\hat{M}}_{\eta }\left(\eta \right)\ddot{\eta }+{\hat{C}}_{\eta }\left(\xi ,\eta \right)\dot{\eta }+{\hat{D}}_{\eta }\left(\xi ,\eta \right)\dot{\eta }+{\hat{g}}_{\eta }\left(\eta \right)=\tau_{\eta }+\Delta f$$ (11)

where,

$$\Delta f=\tau_{d}-\Delta M_{\eta }\left.\left(\eta \right)\right)\ddot{\eta }-\Delta C_{\eta }\left.\left(\xi ,\eta \right)\right)\dot{\eta }-\Delta D_{\eta }\left.\left(\xi ,\eta \right)\right)\dot{\eta }-\Delta g_{\eta }\left(\eta \right)$$ (12)

So that, Eq. 11 can be rewritten as:

$${\hat{M}}_{\eta }\left(\eta \right)\ddot{\eta }+{\hat{N}}_{\eta }\left(\xi ,\eta ,\dot{\eta }\right)=\tau_{\eta }+\Delta f$$ (13)

where,

$${\hat{N}}_{\eta }\left(\xi ,\eta ,\dot{\eta }\right)={\hat{C}}_{\eta }\left(\xi ,\eta \right)\dot{\eta }+{\hat{D}}_{\eta }\left(\xi ,\eta \right)\dot{\eta }+{\hat{g}}_{\eta }\left(\eta \right)$$ (14)

Typically, indoor blimps are equipped with a limited number of actuators to optimize their buoyancy-to-weight ratio. Only essential degrees of freedom are directly controlled, and the possibility of generating lateral thrust is generally eliminated Adamski et al. (2020). As a result, blimps are usually under-actuated systems that are difficult to control. In contrast, the motor positioning for the blimp reference presented in Section 3.1 provides a fully-actuated system that can control all 6 DoF.

Assuming the presence of a motor on the balloon, in Figure 5, the force vector resulting from the motor thrust $f_{p}^{b}$ is defined in a reference frame oriented as *F*_*b*_ and centered at the motor position $P={\left[\begin{array}{ccc} x_{p} & y_{p} & z_{p} \end{array}\right]}^{T}$ with respect to the center of the body frame. To define the three components of the force vector $f_{p}^{b}$ in the *F*_*b*_ frame, the angles *α* and *β* are introduced:

$$f_{p}^{b}=\left[\begin{array}{c} f_{px}\\ f_{py}\\ f_{pz} \end{array}\right]=\left[\begin{array}{c} f_{p}\cos\beta \sin\alpha \\ f_{p}\sin\beta \sin\alpha \\ f_{p}\cos\alpha \end{array}\right]$$ (15)

With respect to the CV pole, the force $f_{p}^{b}$ generates a torque that is equal to the cross product of the position vector and the force vector:

$$c_{p}^{b}=P\times f_{p}^{b}$$ (16)

To build the ***τ*** vector in Eq. 7 the sum of each force and torque component is made:

$$\tau =\left[\begin{array}{c} f_{px,i}+\cdots +f_{px,n}\\ f_{py,i}+\cdots +f_{py,n}\\ f_{pz,i}+\cdots +f_{pz,n}\\ c_{px,i}+\cdots +c_{px,n}\\ c_{py,i}+\cdots +c_{py,n}\\ c_{pz,i}+\cdots +c_{pz,n} \end{array}\right]$$ (17)

with *i* = 1, … , 8.

The $\tau_{d}^{m}$ magnetic disturbance vector presented in Section 2.5 shall be reported from the motor frame to the body frame *R*_*b*_ as follows

$$\tau_{d}^{b}=\left[\begin{array}{c} f_{d}^{b}\\ c_{d}^{b} \end{array}\right]=\left[\begin{array}{c} R_{m}^{b}f_{d}^{m}\\ R_{m}^{b}c_{d}^{m}+P\times f_{d}^{b} \end{array}\right]$$ (18)

where $R_{b}^{m}\left(\alpha ,\beta \right)$ is the inverse of the transformation matrix $R_{b}^{m}\left(\alpha ,\beta \right)$ between the motor frame in Figure 4 and the body frame in Figure 5, defined by the *α* and *β* angles knowing that the force vector $f_{p}^{b^{\prime }}$ has the same direction as the ${\hat{z}}_{m}$ -axis which is the motor rotation axis:

$$R_{b}^{m}\left(\alpha ,\beta \right)=\left[\begin{array}{ccc} \cos\alpha & 0 & -\sin\alpha \\ 0 & 1 & 0\\ \sin\alpha & 0 & \cos\alpha \end{array}\right]\left[\begin{array}{ccc} \cos\beta & -\sin\beta & 0\\ \sin\beta & \cos\beta & 0\\ 0 & 0 & 1 \end{array}\right]$$ (19)

### 3.3 Control system

Controlling LtA vehicles is generally difficult because they are characterized by non-linear dynamics and are affected by air flows, making them a highly coupled class of nonlinear Multi-Input Multi-Output (MIMO) systems Takaya et al. (2006); Yang and Yan (2016). Furthermore, model uncertainties, and external disturbances contribute to the difficulty in maneuvering an airship to track a time-varying reference trajectory Yang et al. (2012).

There are many control system methodologies proposed in the literature for the trajectory tracking problem of blimps such as: Proportional Integrative Derivative (PID) de Paiva et al. (1999); Azinheira et al. (2000), State Feedback Hygounenc and Soueres (2003), Feedback Linearization approach Lee et al. (2005), Back-stepping Control Beji and Abichou (2005) and Adaptive Control Yan et al. (2008). These control systems have the limitations of neglecting dynamic non-linearity and coupling effects considering the linear system, or considering the non-linearities, they do not take into account the uncertainty of the parameters and disturbances. To account for the uncertainties, which are the most critical element in our mission simulation, and the model parameters a robust controller is needed.

The objective of control is to design an algorithm that causes the system to track a desired trajectory

$$\eta_{d}\left(t\right)={\left[\begin{array}{cccccc} x_{d}\left(t\right) & y_{d}\left(t\right) & z_{d}\left(t\right) & \phi_{d}\left(t\right) & \theta_{d}\left(t\right) & \psi_{d}\left(t\right) \end{array}\right]}^{T}$$

such that

$$\|\eta \left(t\right)-\eta_{d}\left(t\right)\|=\|e\left(t\right)\|<\epsilon_{\mathrm{tol}}\;\forall t>t_{\mathrm{conv}}$$ (20)

where ***e*** (*t*) is the tracking error, *t*_*conv*_ < *∞* the convergence time and *ϵ*_*tol*_ the tolerance error.

In this work the trajectory tracking control is simulated partitioning the controller into a model-based portion to linearize and decouple the system and a servo portion to make it fly on the desired trajectory. The system’s parameters appear only in the model-based portion and the servo portion is independent of these parameters. The model-based portion of the control law takes the form

$$\tau_{\eta }={\hat{M}}_{\eta }\left(\eta \right){\tau_{\eta }}^{\prime }+{\hat{N}}_{\eta }\left(\xi ,\eta ,\dot{\eta }\right)$$ (21)

As for the servo law ***τ***_***η***_′, three different strategies have been implemented: the Computed Torque Control (CTC), the Sliding Mode Control (SMC) and the Nonsingular Terminal Sliding Mode Control (NTSMC).

Based on the blimp dynamic Eq. 13, the control law in Eq. 21 represents the control term. It is worth noting that the control law intentionally omits the lumped uncertainty term Δ ***f*** which includes parameter uncertainty and the magnetic disturbances as defind in Eq. 12. This omission leads to the introduction of an external determinitic error into the control system loop, aimed at assessing the robustness of the controller.

#### 3.3.1 Computed Torque Control

The non-linear control technique called CTC is aimed at transforming a non-linear system into a linear one which is then controlled with a classic closed-loop feedback control scheme. This control technique is widely used in robotics Craig (2005); ChaeAn and Hollerbach (1988) and is commonly referred as state feedback linearization. The servo law for the CTC is designed as follows

$$\tau^{\prime }={\ddot{\eta }}_{d}-K_{P}e\left(t\right)-K_{D}\dot{e}\left(t\right)$$ (22)

where, ***K***_*P*_, ***K***_*D*_ are constant positive symmetric matrices and *e*(*t*) as defined in Eq. 20.

#### 3.3.2 Sliding mode control

In the SMC the system is controlled so that it reaches and remain close to a surface, called sliding, which represents the reference of the control system. This technique has been widely used for underwater vehicles and robotics airship Yoerger and Slotine (1985); Bagheri and Moghaddam (2009); Paiva et al. (2009). The servo law for the SMC is defined as follows:

$${\tau_{\eta }}^{\prime }={\ddot{\eta }}_{d}-K_{1}s-K_{2}sat\left(s,\Phi \right)$$ (23)

where, ***K***_1_, ***K***_2_ are constant positive symmetric matrices and ***s*** the sliding surface selected as

$$s\left(t\right)=\dot{e}\left(t\right)+ae\left(t\right)$$ (24)

where $a=\mathrm{diag}\left[\begin{array}{ccc} a_{1} & \cdots & a_{6} \end{array}\right]$ is a constant matrix with *a*_*i*_ > 0 (*i* = 1, … , 6) and ***sat*** (***s***, Φ) is defined ad

$${sat\left(s,\Phi \right)}_{i}=\left\{\begin{array}{c} \frac{s_{i}}{\left|s_{i}\right|}\;\text{if}\;s_{i}>\Phi_{i}\;\\ \frac{s_{i}}{\Phi_{i}}\;\text{if}\;s_{i}\leq \Phi_{i}\; \end{array}\right.$$ (25)

where, Φ_*i*_ is the boundary layer thickness. This particular definition is needed to avoid the problem of chattering due to the high speed switching control law Slotine and Li. (1991).

#### 3.3.3 Non-singular terminal sliding mode control

The continuous SMC can guarantee asymptotic stability of the system in the sliding mode which implies that the system states will converge to the equilibrium point at infinite settling time. In order to further ensure the finite time convergence, a nonlinear sliding mode control called Terminal Sliding Mode Control (TSMC) has been proposed Venkataraman and Gulati (1992) which has been applied to the trajectory tracking of robotics airship Yang (2018). Compared to conventional SMC, TSMC provide fast and finite time convergence and high precision control. However, it sufferers of singularity problems Feng et al. (2001). To avoid the singularity problem, the NTSMC has been developed Feng et al. (2013); Chen and Lin (2011) and successfully applied for autonomous underwater vehicles and robotic airships Londhe et al. (2017); Yang and Yan (2016); Yang et al. (2012); Yang (2018) The servo law for the NTSMC is defined as

$${\tau_{\eta }}^{\prime }={\ddot{\eta }}_{d}-\beta \frac{q}{p}\dot{e}{\left(t\right)}^{\left(2-\frac{p}{q}\right)}-\zeta \mathrm{sign}\left(s\right)$$ (26)

where $\zeta =\mathrm{diag}\left[\begin{array}{ccc} \zeta_{1} & \cdots & \zeta_{6} \end{array}\right]$ is a diagonal matrix with *ζ*_*i*_ > 0, *p* and *q* are positive odd integers with *p* > *q*, $\beta =\mathrm{diag}\left[\begin{array}{ccc} \beta_{1} & \cdots & \beta_{6} \end{array}\right]$ with *β*_*i*_ > 0 (*i* = 1, … , 6) and the nonlinear sliding surface ***s***

$$s\left(t\right)=e\left(t\right)+\frac{1}{\beta }\dot{e}{\left(t\right)}^{\frac{p}{q}}$$ (27)

## 4 Results and discussion

**FIGURE 6:**
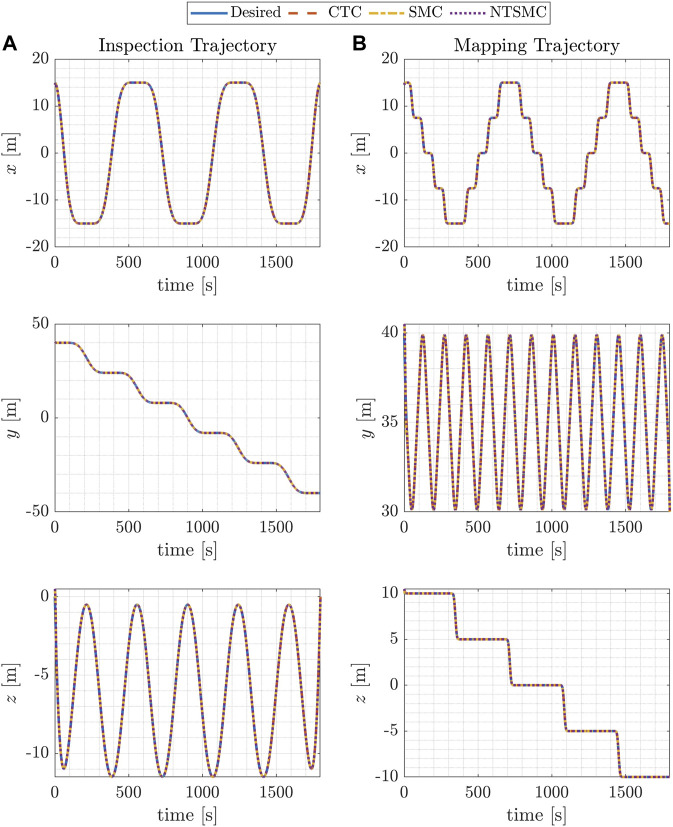
Blimp coordinates for the desired and controlled trajectories using three different control methods. **(A)** displays the results of the inspection trajectory, while **(B)** shows those of the mapping trajectory.

**FIGURE 7:**
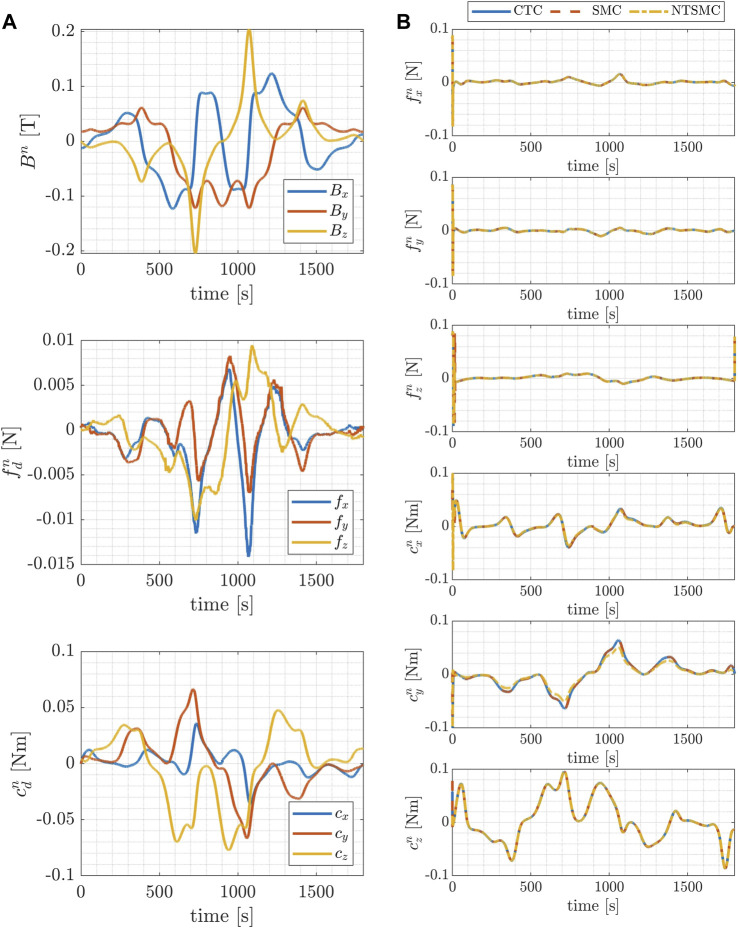
Inspect trajectory: time evolution of the components of the magnetic field, the magnetic force and the magnetic torque acting on the blimp, during the desired trajectories in the inertial reference frame **(A)**; time evolution of the control forces and torques exerted by the blimp’s motors during the desired trajectories in the inertial reference frame **(B)**.

**FIGURE 8:**
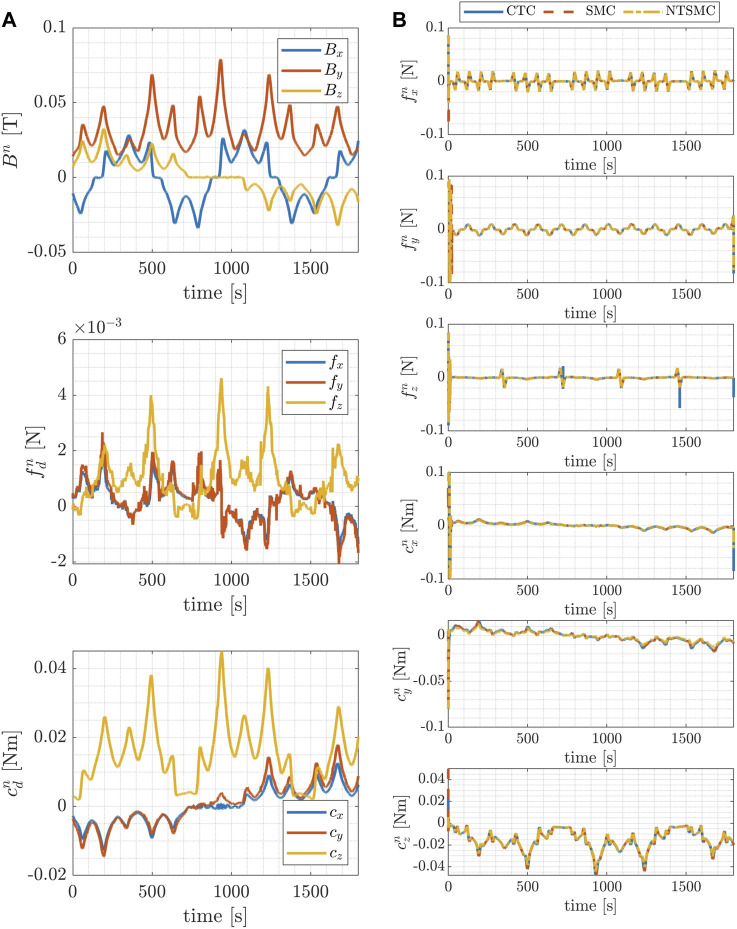
Mapping trajectory: time evolution of the components of the magnetic field, the magnetic force and the magnetic torque acting on the blimp, during the desired trajectories in the inertial reference frame **(A)**; time evolution of the control forces and torques exerted by the blimp’s motors during the desired trajectories in the inertial reference frame **(B)**.

**FIGURE 9:**
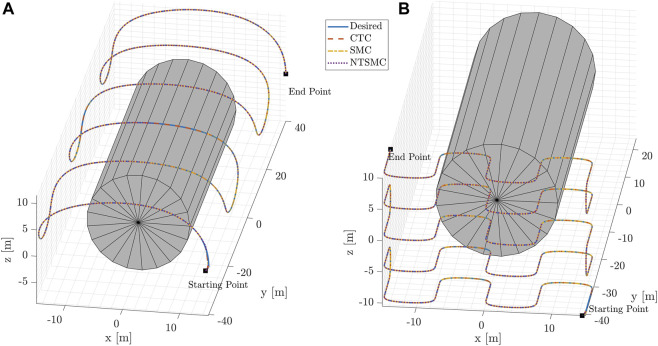
3D trajectories (desired + controlled) for the inspect case **(A)** and mapping case **(B)**. The grey cylinder is a rough representation of the particle detector.

The effectiveness of the proposed control schemes have been verified by performing numerical simulation using MATLAB^®^ tool. The magnetic field map was obtained from a CST Studio Suite simulation of the FCC-hh detector as explained in Section 2.3. The efficacy of the proposed controllers has been evaluated with the lumped uncertainty term Δ ***f*** defined in Eq. 12 which comprises a 20% uncertainties in the model parameters, and the external disturbance defined in Eq. 18 with *m*_*p*_ = 0.5 Am^2^ and *m*_*c*_ = 0.1 Am^2^. The lumped uncertainty term is added externally to the control law and the dynamics of the blimp. This setup ensures that during the integration of the dynamics to derive the blimp’s state, the lumped term acts as an external deterministic disturbance. The trajectory tracking control was simulated using the variable step Runge–Kutta integrator. The following nominal controller settings were considered for CTC: ***K***_*P*_ = 50*I*_6_, ***K***_*D*_ = 100*I*_6_; SMC: ***K***_1_ = 1*I*_6_, ***K***_2_ = 2*I*_6_, ***a*** = 1*I*_6_, Φ = 0.5 and NTSMC: *p* = 99, *q* = 97, *β* = *I*_6_, *ζ* = *I*_6_ and Φ = 0.5. The experiment results for both trajectory types are presented in Figures 6–9.

Figure 6 show the time evolution of the x, y, and z coordinates of the blimp’s position for the desired trajectories, employing three different control methods. The left-hand column presents outcomes for the inspection trajectory, while the right-hand column showcases those for the mapping trajectory. The presented results indicates the blimp’s capability to follow the desired trajectories with some deviations. Notably, the magnitude of these deviations varies across the control methods.

Figure 7 and Figure 8 present, in the left-hand column, the temporal evolution of magnetic field components, magnetic force, and magnetic torque acting upon the blimp during the desired trajectories within the inertial reference frame. These results pertain to both the inspection case (Figure 7) and the mapping case (Figure 8). The trends of magnetic forces and torques illustrate the term ***τ***_***d***_, encapsulated within the lumped uncertainties term Δ ***f***. These plots show how the disturbance varies as the blimp navigates through the cavern. The specific pattern observed in the magnetic disturbance ***τ***_***d***_ originates as a result of the motor’s modeling, which is conceptualized as a magnetic dipole moving within space and composed of two distinct components: a stationary contribution (the motor’s permanent magnet) and another component that aligns with the magnetic field lines (the motor’s cover). This dynamic interplay leads to fluctuations in the total moment, resulting in localized forces and torques exerted on the blimp at different positions within the field. Moreover, it is noteworthy that the disturbance magnetic torques exhibit a greater magnitude compared to the disturbance magnetic forces across both types of trajectories. This observation implies that the blimp is predominantly subjected to higher disturbance torques rather than disturbance forces.

Additionally, on the right-hand column of Figure 7 and Figure 8 the time evolution of the control forces and torques respectively exerted by the blimp’s motors during the desired trajectories in the inertial reference frame is represented. The magnitude of the control forces is shown to remain within the predetermined range of 0.1 N for both trajectory types, indicating that the control system is able to maintain the desired trajectory without exceeding the maximum allowable force.

Finally, Figure 9 displays the two type of mission scenarios through the 3D trajectories of the blimp with a simplified representation of the detector used in the experiment. On the left-hand side the inspection trajectory and on the right-hand side the mapping trajectory show that the blimp successfully navigates around the detector while inspecting and mapping the surrounding area with all the three control techniques.

The simulation results demonstrate the effectiveness of the proposed control methods in enabling the blimp to navigate through a complex environment while maintaining stability and accuracy. The blimp is able to follow the desired trajectory with some deviation, and the magnitude of the deviation varies depending on the control method used. The use of a cylindrical detector in the center of the trajectory helps to visualize the location of the target and assess the accuracy of the blimp’s trajectory. The NTSMC algorithm outperforms the CTC and SMC controllers in terms of smooth transient response, convergence of tracking errors to zero in less than 2 s, and robustness against magnetic disturbance and model parameter uncertainty.

In the present study, various Model-Based Control (MBC) methodologies have been examined and compared, with a focus on well-known modern control approaches such as CTC, SMC, and NTSMC. These techniques have showcased notable effectiveness in trajectory tracking for the blimp within the CERN environments. However, one of the primary challenges of MBC techniques is that they heavily rely on the accuracy of the mathematical model used to represent the system. Even for very simple cases, the mathematical modeling always introduces numerous sources of uncertainty such as model parameters uncertainties, unmodeled dynamics, and the presence of disturbances and noise.

Modern control methodologies, designed to address these challenges, face certain critical issues, particularly when dealing with highly nonlinear complex systems like UAVs Gu et al. (2019). For instance, controller complexity escalates with system order. These challenges highlight the need for innovative and intelligent control design methods that ensure stability, adaptability, and efficiency in the face of various uncertainties. As a result, contemporary research in robust and adaptive control has shifted towards systems that include rule-based, knowledge-based, and learning-based techniques Santoso et al. (2018). These advances involve using methods such as fuzzy logic systems Shi et al. (1999) and artificial neural networks (ANNs) Abraham (2001).

Among these approaches, for example, ANNs hold promise for mathematical model identification and controller design, due to their ability to autonomously adapt and learn in real time complex dynamics, with relatively simple hardware computation and implementation. To achieve this, ANNs, however, require substantial amounts of training data, and one must also consider their susceptibility to learning spurious relationships that hinder generalization, their lack of interpretability due to their black-box nature, and the absence of systematic approaches for designing ANN architectures. Nevertheless, to address these challenges, a complementary relationship between MBC techniques and ANNs becomes evident. The explicit knowledge of the system underlying MBC can aid the training process and enhance the performance and interpretability of ANNs Gu et al. (2019).

## 5 Conclusion and future work

This study has demonstrated the feasibility of inspection and mapping missions for indoor blimps in the harsh environments of CERN’s particle detectors. By developing a robust controller, it was possible to achieve accurate trajectory following even in the presence of magnetic disturbances and uncertainty in the estimation of dynamic parameters. The use of a cylindrical detector in the center of the trajectory proved useful for visualizing the location of the target and assessing the accuracy of the blimp’s trajectory.

Moreover, the results of this study suggest that the development of control systems for flying robots in underground areas such as CERN can provide a promising approach for the inspection and surveillance of particle accelerator facilities. The blimps could be used to quick access the experimental detector caverns at any moments for inspections, also during beam run, when, usually, due to the high radiation level and other safety reasons, no personnel is allowed in the caverns. Thus, they would reduce the risk of radiation exposure for human personnel and minimize the detector shout down time by optimizing the response time of the surveillance and warning system.

While this study primarily focused on high levels of magnetic fields present in CERN’s underground experimental areas, the discussed system can also be applied to other challenging environments. By adapting the findings of this study, it becomes possible to utilize indoor blimps for inspection and mapping tasks in various settings, including those with lower disturbance levels as for instance indoor industrial environments.

Further investigations are needed to improve the accuracy of the blimp’s control system and to test the magnetic disturbance model. To this end, CERN is planning to carry out a campaign of electromagnetic measurements on electrical motors in the near future. The aim is to gain a deeper understanding of the magnetic interaction between the ferromagnetic materials within the motor and the surrounding environment. This study will encompass phenomena like motor hysteresis and the impact of field frequency on motor performance. Regarding the blimp’s dynamics and control within uncertain environments, the combination of data-driven techniques alongside model-based approaches would enhance controller effectiveness, facilitating a more comprehensive characterization of the nonlinear characteristics of magnetic disturbances.

In summary, this study demonstrates the theoretical feasibility of using indoor blimps for inspection and mapping missions in magnetic field environments such as the future CERN underground experimental areas. These systems could provide a safer and more efficient approach to the inspection and surveillance of particle accelerator facilities by providing access to unreachable areas for humans and reducing the detector downtime.

## Data Availability

The raw data supporting the conclusion of this article will be made available by the authors, without undue reservation.

## Footnotes

1
https://home.cern/

2
https://home.cern/science/accelerators/large-hadron-collider

3
https://atlas.cern/Discover/Detector

4
https://alice-collaboration.web.cern.ch/

5
https://cms.cern/detector

6
http://lhcb.web.cern.ch/

7 The current CERN detector cavern environment can be virtually explored using Google Street View technology https://home.cern/news/news/cern/visit-cern-sites-new-google-street-view.

8
https://ep-dep-dt.web.cern.ch/

9
https://ep-dep.web.cern.ch/rd-experimental-technologies

10
https://fcc.web.cern.ch/

11
https://https://fcc-cdr.web.cern.ch/

12
https://ch.mathworks.com/

13
https://www.3ds.com/products-services/simulia/products/cst-studio-suite/

14
https://www.bitcraze.io/products/crazyflie-2-1/
